# Why netizens rebut transnational fake news: examining the role of perceived fake news seriousness, emotional response, and information-seeking motivation

**DOI:** 10.3389/fpsyg.2026.1852890

**Published:** 2026-10-19

**Authors:** Yanjun Lin, Yuge Xu, Zhihui Ying

**Affiliations:** State Key Laboratory of Media Convergence and Communication, Communication University of China, Beijing, China

**Keywords:** emotional response, information-seeking motivation, perceived fake news seriousness, rebuttal, transnational fake news, anxiety, anger

## Abstract

**Introduction:**

In today’s globalized media environment, transnational fake news has become a tool of cognitive warfare, provoking strong public responses. Despite growing attention to disinformation governance, few studies have unpacked the psychological and emotional drivers of netizens’ voluntary rebuttal of transnational fake news. This study aims to fill this gap by examining how the perceived seriousness of fake news affects users’ rebuttal behaviors via emotional media-tors—anger and anxiety.

**Methods:**

Drawing on the third person effect, this study constructed a moderated mediation model with anger and anxiety as emotional mediators and information seeking motivation as moderator. A survey of 309 Chinese netizens aged 16 to 45 was conducted, and mediation and moderation analyses were applied to test the research hypotheses.

**Results:**

Mediation analysis confirmed the significant roles of both emotions, while moderation analysis showed that information seeking motivation strengthens the link between anxiety and rebuttal, but not between anger and rebuttal. The findings emphasize the significance of user-centered approaches in combating transnational disinformation.

**Discussion:**

By clarifying the psychological and emotional mechanisms behind rebuttal behaviors, this study offers insights for designing more effective public communication strategies in the digital age.

## 1 Introduction

In the globalized media environment, fake news has increasingly become a strategic instrument of cognitive warfare, shaping public sentiment and disrupting transnational information flows (Vasu et al., 2018). Recent international conflicts—such as the Russia–Ukraine war and the Israel–Iran confrontation—have illustrated how manipulated content quickly crosses borders and reshapes international interpretations of another country’s policies, actions, or domestic conditions (Hameleers, 2025; Johansson-Nogués and Şimanschi, 2023). Such dynamics highlight that fake news is no longer a localized communication problem but a cross-national cognitive challenge that affects how countries are perceived globally.

Against this backdrop, the conceptualization of fake news has evolved beyond factual falsity. Early research often understood fake news primarily in terms of factual inaccuracy (Tandoc et al., 2018). However, the fast-moving and multi-platform nature of contemporary information diffusion has complicated this understanding (Vosoughi et al., 2018). Information is frequently reposted, reframed, and detached from its original context, obscuring both its source and intended purpose. These complexities have prompted scholars to define fake news not only by factual inaccuracy but also by its capacity to erode trust in information credibility and distort public discourse (Wardle and Derakhshan, 2017; Deluggi and Ashraf, 2023). This effect-oriented understanding is particularly relevant in global communication, where information asymmetries mean that foreign audiences often rely on limited cues to interpret another country’s affairs.

Correspondingly, the governance of fake news has also become more complex. In response to the increasing difficulty of identifying and controlling misinformation in digital environments, many scholars have turned to technological approaches. Big data analytics have been applied to context-based fake news detection by integrating large-scale information processing, contextual cues, and behavioral patterns to improve identification accuracy in digital environments (Shahzad et al., 2022). Similarly, AI-based approaches, including machine learning and automated classification techniques, have created new possibilities for monitoring misinformation at scale (Iqbal et al., 2025). These studies demonstrate that fake news research has moved beyond content-level verification toward a broader concern with the technological and infrastructural conditions under which misinformation is detected, disseminated, and controlled.

Yet detecting fake news is not the same as reducing its influence. Once misleading information enters social media networks, its effects are shaped not only by whether it can be identified as false, but also by how users encounter, interpret, share, and contest it (Sun and Xie, 2024). Systematic reviews of fake news diffusion and diffusion control have emphasized that effective governance involves multiple layers, including detection, prevention, user intervention, corrective communication practices, and users’ media literacy in recognizing and resisting misleading information (Khan et al., 2022; Shahzad et al., 2023; Shahzad and Khan, 2024). In this sense, ordinary users are no longer passive recipients of misinformation but active participants who may contribute to either the amplification or correction of false narratives.

For domestic users, the possibility that foreign publics—or actors engaged in a bilateral or multilateral dispute—may misinterpret national issues or ongoing international matters can heighten concerns that “others” are more vulnerable to misinformation. Such perceptions are consistent with the third-person effect (TPE), which suggests that individuals tend to perceive media influence as stronger on others than on themselves (Davison, 1983). In transnational fake news contexts, these concerns may escalate into anxiety about national image distortion or anger toward perceived attempts to mislead foreign audiences, thereby motivating users to challenge false narratives in online spaces. Recent studies have shown that user-driven rebuttal can complement professional fact-checking and shape the discursive contestation of false narratives (Saeed et al., 2022; Panizza et al., 2023). Yet existing research has not fully explained how perceptions of fake news seriousness translate into rebuttal behavior, nor how emotional responses such as anger or anxiety shape this process within highly politicized transnational contexts.

This issue is particularly noteworthy in the context of China. China-related issues have increasingly become contested topics in global information flows, where misleading or decontextualized information may affect how foreign publics interpret China’s policies, actions, and social conditions (Vasu et al., 2018; Waisbord, 2018). Meanwhile, China has the world’s largest online population, with 1.125 billion netizens according to the 57th Statistical Report on China’s Internet Development (CNNIC, 2026). As cross-national misinformation becomes increasingly prevalent and news consumption becomes more globalized, this vast online population holds significant potential as a corrective force in combating misleading narratives. Therefore, understanding the psychological mechanisms underlying rebuttal behaviors is essential for explaining how individuals participate in the governance of transnational fake news.

Based on the third-person effect (TPE), the current study aims to examine how the perceived seriousness of fake news—including misinformation about domestic issues and contested international matters—affects rebuttal behaviors, particularly comments or shares countering disinformation about one’s home country. It examines anger and anxiety as two discrete emotional mediators between perceived fake news seriousness and rebuttal behavior (Horner et al., 2023). The study also investigates how information seeking motivation, a key factor in personal involvement (Steensen et al., 2020; Bhagat and Kim, 2023; Omar et al., 2024), moderates this process. This study adopts a user-centered cognitive-affective perspective, which offers a novel approach to improving the governance of transnational fake news and formulating more effective rebuttal strategies.

## 2 Theoretical hypothesis

### 2.1 The third-person effect and user responses to transnational fake news

The concept of fake news has evolved substantially as information flows have become increasingly global and complex. Earlier definitions tended to equate fake news with false or fabricated information, emphasizing factual inaccuracy as the central criterion (Tandoc et al., 2018). Later distinctions—such as the differentiation between misinformation (false content without intent to harm) and disinformation (false content created with manipulative intent)—provided greater conceptual nuance (Wardle and Derakhshan, 2017). Yet in transnational communication environments, determining intent or identifying the original source of a message is often difficult, as information is rapidly reposted, reframed, and detached from its initial context. For this reason, recent scholarship increasingly supports an effect-oriented definition, viewing fake news as information that undermines trust in communicative credibility, distorts public discourse, or facilitates strategic misinterpretations regardless of the producer’s intent (Phiri, 2025).

These definitions become especially salient in transnational situations. International audiences typically possess limited contextual knowledge about foreign societies, policy processes, or cultural practices, making them more reliant on simplified cues when interpreting unfamiliar events (Waisbord, 2018). In such contexts, misleading content can easily shape perceptions of a country’s actions, intentions, or values. Moreover, in situations involving diplomatic disagreements, geopolitical tensions, or contested international issues, false or distorted narratives may be produced and circulated by different actors seeking to position themselves advantageously (Seib, 2021). Accordingly, transnational fake news can be understood as misleading, decontextualized, or strategically framed information that affects how one country is interpreted by external publics or by other actors involved in international affairs.

Under this effect-oriented understanding, fake news governance research has increasingly examined not only factual identification and detection but also how misinformation spreads across digital platforms and how its influence can be controlled. Studies on fake news diffusion show that its circulation is closely related to users’ cognitive judgments, emotional states, perceived relevance, and social interactions (Shahzad et al., 2023). These findings imply that users’ responses are shaped by their interpretations of who may be affected and how serious the consequences may become. However, limited attention has been paid to how such interpretations translate into corrective or rebuttal behaviors, especially in transnational contexts. When misleading narratives may influence external publics’ interpretations of a country’s actions, intentions, or values, domestic users may perceive a need to challenge them to prevent national image distortion or international misunderstanding. Thus, a key unresolved question is how domestic users perceive the influence of transnational fake news on external publics and how such perceptions translate into corrective or rebuttal behaviors.

The third-person effect (TPE), originally proposed by Davison (1983), offers a useful framework for understanding public responses to such phenomena. TPE research consistently shows that individuals tend to believe that harmful or undesirable messages exert greater influence on others than on themselves. This perceptual gap has been demonstrated across diverse media contexts and supported by insights from social categorization theory, the elaboration likelihood model, and attribution theory (Stenbjerre, 1997; White, 1997; Paul et al., 2000). Importantly, the effect is amplified when messages are ambiguous, socially negative, or potentially threatening conditions that align closely with the characteristics of transnational fake news.

In the realm of international communication, TPE is particularly relevant because individuals frequently assume that external audiences or out-groups lack adequate contextual knowledge about their home country (Park et al., 2012). As a result, they may judge these groups to be more susceptible to misleading narratives about national issues, foreign policy decisions, or international disputes. Prior studies also show that stronger third-person perceptions can shape attitudes toward information governance and corrective interventions (Sun et al., 2008), indicating that TPE influences both cognitive appraisals and normative judgments about how misinformation should be managed. These insights provide a theoretical foundation for examining how people perceive the societal impact of transnational fake news and why they may develop heightened concern about its influence on others.

### 2.2 Perceived fake news seriousness and emotional activation

Perceived fake news seriousness refers to a user’s cognitive evaluation of the potential harm posed by misleading information—whether through its threat to public understanding, national reputation, or the integrity of transnational discourse (Bhagat and Kim, 2023). Unlike simple disbelief or perceived inaccuracy, seriousness captures an appraisal of consequentiality, reflecting a judgment that misinformation can produce meaningful social, political, or ethical damage. As such, it functions as a higher-order cognitive assessment that shapes how individuals interpret the implications of misinformation.

Appraisal theories of emotion posit that emotional responses arise not from the stimulus itself but from cognitive evaluations of its meaning and implications (Lazarus, 1991; Lerner and Keltner, 2000). These evaluations involve assessments of certainty, controllability, responsibility, and potential outcomes. When individuals judge fake news as serious, they evaluate its implications across these dimensions, making emotional activation more likely. Different appraisal patterns, in turn, give rise to different emotional profiles.

Anxiety is associated with appraisals characterized by uncertainty, low controllability, and anticipation of negative consequences (Freiling et al., 2023). When misinformation is perceived as serious, users may anticipate harmful outcomes—such as worsening misperceptions abroad or reputational damage—that they feel unable to prevent. This combination of uncertainty and low control enhances anxious arousal.

Anger, in contrast, is linked to appraisals involving responsibility, blame, and perceived injustice (Horberg et al., 2011). If serious misinformation is attributed to irresponsible, biased, or intentionally misleading actors (e.g., foreign media, or politically motivated groups), users may interpret the content as a moral or strategic violation. The more serious the misinformation is judged to be, the more intense the perceived wrongdoing is, thereby amplifying anger.

Accordingly, the following hypotheses are proposed:

*H1a*: Perceived fake news seriousness is positively associated with anxiety.

*H1b*: Perceived fake news seriousness is positively associated with anger.

### 2.3 Emotional responses and transnational fake news rebuttal

Emotional responses play a central role in shaping communicative behavior in digital environments, particularly when misinformation pertains to politically sensitive or culturally salient national issues. In misinformation contexts, emotions can affect how individuals process information, evaluate credibility, and decide whether to engage with corrective communication (Martel et al., 2020). Once emotional arousal occurs, it may serve as a proximal motivator of behavior, shaping how individuals engage with threatening or misleading content. Among the spectrum of emotions, anger and anxiety are two of the most frequently studied in misinformation research; yet they lead to distinct behavioral outcomes.

Anger is typically conceptualized as an approach-oriented emotion linked with certainty, high perceived control, and blame attribution (Lerner and Tiedens, 2006). These appraisals make individuals more inclined to confront the source of misinformation, correct perceived inaccuracies, or engage in assertive communicative acts. Such tendencies may be especially salient in transnational contexts, where individuals perceive external actors—such as foreign media or political groups—as responsible for disseminating misleading narratives about their home country.

Anxiety, in contrast, is an avoidance-oriented emotion associated with uncertainty, perceived lack of control, and heightened risk perception (Carleton, 2016). These appraisals motivate behaviors aimed at reducing ambiguity, such as seeking additional information, consuming corrective content, or sharing fact-checks to restore a sense of informational stability. Even if less confrontational than anger-driven responses, anxiety can still encourage engagement with rebuttal efforts when individuals perceive misinformation as having potentially serious societal or reputational consequences.

This differentiation is important for understanding users’ motivations to rebut transnational fake news. Rebuttal behaviors—including corrective commenting, counter-narrative posting, or sharing content with clarifying intent—can stem from different emotional orientations. Individuals experiencing anger may feel compelled to actively defend national identity or challenge perceived wrongdoing, while those experiencing anxiety may rebut misinformation as a means of mitigating uncertainty or safeguarding the national information environment. Therefore, we hypothesize:

*H2a*: Anxiety is positively associated with transnational fake news rebuttal.

*H2b*: Anger is positively associated with transnational fake news rebuttal.

According to the third-person effect, individuals often gauge the severity of transnational fake news by its perceived impact on others. This perceptual gap reinforces their overall assessment of the severity. Appraisal theories of emotion further explain how this drive is expressed through emotional arousal. Specifically, perceived threats to others may elicit either anxiety or anger depending on users’ appraisal of uncertainty or injustice. While these emotions differ in orientation, both can strengthen users’ intention to directly rebut fake news in public forums. That is, anxiety may prompt users to engage in rebuttal to regain clarity and control, while anger may compel users to confront perceived wrongdoing. Thus, both emotions serve as affective mechanisms that translate the cognitive assessment of impact on others into personal rebuttal behaviors. Therefore, we hypothesize:

*H3a*: Anxiety mediates the relationship between perceived fake news seriousness and rebuttal behavior.

*H3b*: Anger mediates the relationship between perceived fake news seriousness and rebuttal behavior.

### 2.4 Information-seeking motivation and emotional responses

According to the third-person effect, people tend to believe that media information has a greater impact on others than on themselves. This gap in perceived influence is often more pronounced in controversial transnational fake news. Departing from the traditional pragmatic orientation toward information-seeking motivation, recent scholarship suggests that such motivations are significantly shaped by one’s personal and environmental context (Shen et al., 2024). Human behavioral patterns fluctuate across varying psychological states. Specifically, the capacity for different emotions to stimulate distinct information-seeking motivations has been established (Coan et al., 2021), with motivations driven by negative affect and discomfort remaining predominant (Savolainen, 2014). For instance, when individuals experience anxiety or anger triggered by transnational fake news, they may develop an intent to rebut such information. However, emotional arousal does not universally translate into action; the intensity of information-seeking motivation dictates the specific behavioral patterns used to counter misinformation. Individuals with higher information-seeking motivation are more likely to utilize emotions, such as anxiety and anger, as triggers for purposeful action rather than passive discomfort. In contrast, individuals low in information-seeking motivation may remain in states of unresolved anxiety or diffuse anger, lacking the volitional drive to convert emotional discomfort into corrective engagement (Liao and Fu, 2013; Zhou and Shen, 2024). Instead, these individuals are more prone to adopt information-avoidance behaviors.

These variations in information seeking motivation significantly influence how users interpret and respond to affectively charged content, such as transnational fake news. Underlying this influence is self-efficacy, a critical cognitive resource that “influences the relationships between information-processing variables and cognitive outcomes” (Lo et al., 2013). Research indicates that self-efficacy plays a moderating role between information-seeking motivation and cognition (Brown et al., 2001). Individuals with high information-seeking motivation typically possess greater self-efficacy, leading them to believe more firmly in their capacity to manage situations effectively through information acquisition and processing.

While anxiety is typically associated with uncertainty and risk aversion, individuals with a high motivation to seek information may view the situation as controllable through engagement, thereby facilitating a transition from avoidance to action. Similarly, while anger naturally evokes approach tendencies, this behavioral manifestation tends to be more pronounced in individuals with strong information-seeking motivation (Shen et al., 2024). In this way, information-seeking motivation operates as a moderator in the emotion–behavior pathway.

Accordingly, we propose the following hypotheses:

*H4a*: Information-seeking motivation positively moderates the relationship between anxiety and transnational fake news rebuttal.

*H4b*: Information-seeking motivation positively moderates the relationship between anger and transnational fake news rebuttal.

*H5a–b*: Information-seeking motivation moderates the indirect effect of perceived fake news seriousness on transnational fake news rebuttal via (a) anger and (b) anxiety, such that the higher the motivation, the stronger the indirect relationship.

As shown in Figure 1, this study develops a proposed structural model to examine all hypotheses.

**Figure 1:**
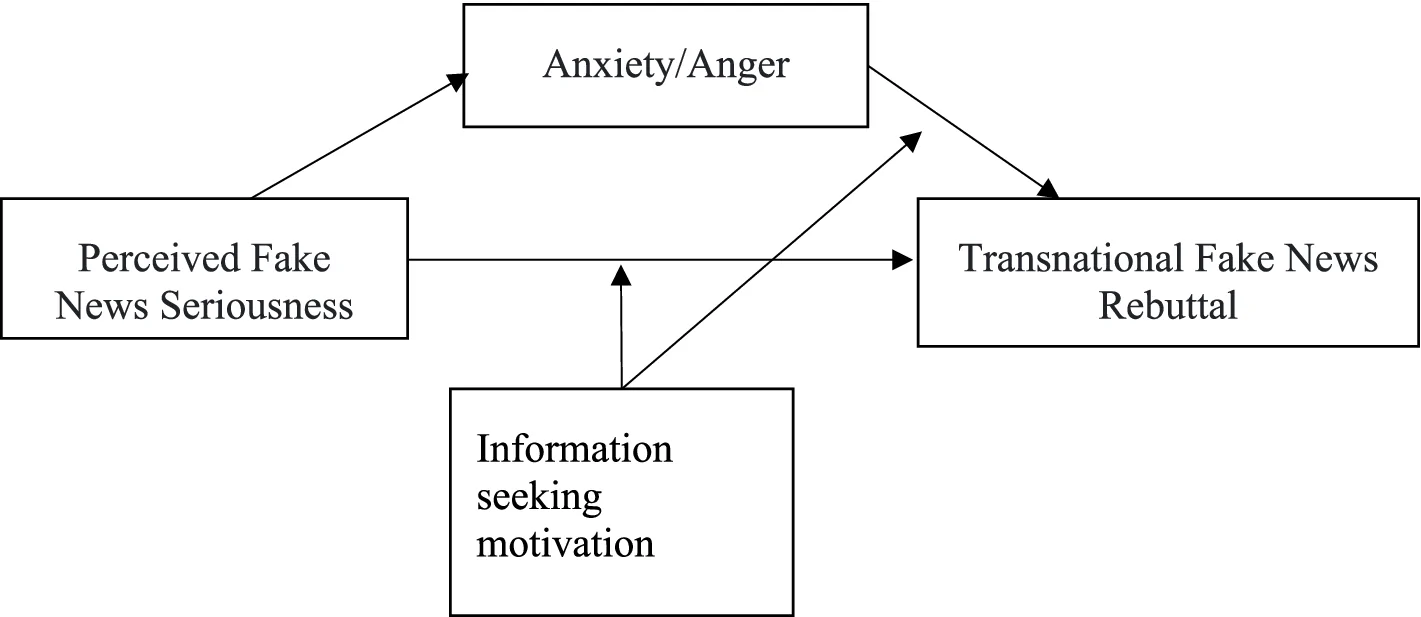
Proposed research model with all hypotheses.

## 3 Data and methods

### 3.1 Procedures

This study commissioned Changsha Ranxing Information Technology Co., Ltd. to issue the official questionnaire via its Wenjuanxing platform. Data collection occurred from October 9 to 18, 2024, with a final sample of 309 participants (50% female, mean age 32, SD = 8.04), all of whom regularly read transnational news. The online survey targeted internet users aged 16 to 45, reflecting the demographic distribution of the 47th National Census (CNNIC, 2021). We chose the 2021 report because there has been no publicly available data indicating the age and regional distribution over the past 4 years (2022–2025). The urban–rural ratio (townships, towns, and others considered rural) is 7:3; 11.27% were aged 16–19, 27.98% were between 20 and 29 years, 39.99% were aged 30–39, and 20.76% were between 40 and 45 years. Anonymity was ensured, and informed consent was obtained prior to the survey.

### 3.2 Measures

#### 3.2.1 Transnational fake news rebuttal

To measure rebuttal behaviors of transnational fake news, we included 3 items to indicate users’ behavioral tendency to rebut transnational fake news. For example, “I would post a comment to inform others that this is fake news”; “I would share a news article that corrects this piece of fake news.” These items were assessed on a seven-point scale, ranging from “strongly disagree” to “strongly agree” (Cronbach *α* = 0.763) (Chang, 2021).

#### 3.2.2 Perceived fake news seriousness

Perceived fake news seriousness originated from the perceived severity of misinformation scale (Xiao, 2024), suggesting that perceived fake news seriousness is instrumental. The original scale included three items in the context of COVID-19-related misinformation combat, therefore, the health-related item was removed as it was not relevant to this study. The final scale consists of two items. The items were, “I believe that being influenced by misinformation has serious negative consequences for me/others”; “I believe that being influenced by misinformation is extremely harmful to me/others,” again on a seven-point scale, ranging from “strongly disagree” to “strongly agree” (Cronbach’s *α* = 0.720).

#### 3.2.3 Anxiety/anger

The measures used for the emotion aroused by transnational fake news were adapted from Watson et al. (1988). The construct of anxiety was measured using two items: nervous and jittery, while anger was assessed through the items hostile and irritable. For example, “When encountering fake news from transnational media that contradicts your understanding of the news, to what extent do you experience the following emotions?” was rated by participants on a 7-point scale, ranging from “Not experienced at all” (Cronbach’s α = 0.770) to “Very strongly” (Cronbach’s α = 0.724).

#### 3.2.4 Information-seeking motivation

The moderator variable for this study was measured by Lee (2013), the 7-point Likert-type scale consisted of one item “the reason for consuming transnational news is to obtain important new information,” which ranged from “strongly disagree” to “strongly agree.”

Given that age, gender, and educational level are commonly selected as control variables fake news research (Guess et al., 2019; Rampersad and Althiyabi, 2020; Knuutila et al., 2022), we controlled these demographic variables in the present study.

### 3.3 Data analysis

Descriptive statistics and a correlation matrix were first generated, followed by testing mediation and moderation effects using the PROCESS macro (Hayes, 2012). Model 4 was employed to assess the mediation of anxiety/anger between perceived fake news seriousness and transnational fake news rebuttal, and Model 14 was used to examine moderation by information-seeking motivation.

Bootstrapping with 5,000 samples and 95% bias-corrected confidence intervals (CIs) ensured the significance of indirect effects (Hayes and Scharkow, 2013). The results indicated that these effects were significant when the CIs excluded zero.

## 4 Results

### 4.1 Descriptive and correlation analysis

Descriptive statistics (means, standard deviations, and zero-order correlations) were generated for all the variables and are presented in Table 1. The internal consistency alphas are also shown in the table. The results showed that the correlation between perceived fake news seriousness and transnational fake news rebuttal was positive.

**Table 1:** Descriptive statistics, alpha coefficients, and correlations.

Variables	M	SD	1	2	3	4	5	6	7	8
1. Gender	—	—	—	—	—	—	—	—	—	—
2. Age	31.870	8.04	−0.095	—	—	—	—	—	—	—
3. Education	3.850	0.620	0.063	0.060	—	—	—	—		—
4. Serious	5.634	0.970	−0.082	−0.052	0.014	(0.720)	—	—	—	—
5. Anxiety	3.898	1.505	0.036	0.042	−0.132*	0.177**	(0.770)	—	—	—
6. Anger	4.138	1.563	−0.076	0.044	−0.039	0.169**	0.472**	(0.724)	—	—
7. Rebuttal	4.246	1.386	−0.033	0.134*	−0.001	0.192*	0.249**	0.250**	(0.763)	—
8. Information-seeking motivation	5.630	1.081	−0.089	0.058	−0.067	0.182**	0.249**	0.213*	0.286**	—

*N* = 309. Internal reliabilities (alpha coefficients) for the constructs are given in parentheses on the diagonal. ISM, information seeking motivation; *p < 0.05, **p < 0.01.

### 4.2 Mediation analysis

According to Model 1 (Table 2), perceived fake news seriousness was positively related to anxiety (B = 0.188, SE = 0.056, *p* < 0.001), and anger (B = 0.168, SE = 0.057, *p* < 0.01). Hence, Hypothesis 1a/b was supported. According to Model 2 (Table 2), anxiety/anger was positively associated with transnational fake news rebuttal (B = 0.149, SE = 0.063, *p* < 0.001; B = 0.150, SE = 0.062, *p* < 0.01). Therefore, Hypothesis 2a/b was supported.

**Table 2:** Testing the mediation effect of anxiety/anger on the relationship between perceived fake news seriousness and transnational fake news rebuttal.

Predictors	Model 1 (anxiety)		Model 2 (anger)		Model 3 (rebuttal)	
	B (SE)	*t*	B (SE)	*t*	B (SE)	*t*
Age	0.666 (0.562)	1.186	0.050 (0.057)	0.874	0.127 (0.055)	2.332 *
Gender	0.133 (0.112)	1.184	−0.109 (0.114)	−0.963	−0.008 (0.110)	−0.073
Education	−0.231 (0.090)	−2.564**	−0.066 (0.091)	−0.724	0.024 (0.088)	0.268
Seriousness	0.188 (0.056)	3.354***	0.168 (0.057)	2.966**	0.146 (0.056)	2.637**
Anxiety	—	—	—	—	0.149 (0.063)	2.382**
Anger	—	—	—	—	0.150 (0.062)	2.421**
R2	0.057	—	0.036	—	0.120	—
F	4.626**	—	2.867**	—	6.848*	—

*N* = 309. Each column is a regression model that predicts the variable at the top of the column; *p < 0.05, **p < 0.01, ***p < 0.001.

Regarding the path among perceived fake news seriousness, anxiety, and transnational fake news rebuttal, the results showed that perceived fake news seriousness had an indirect effect on transnational fake news rebuttal (B = 0.028). Bootstrapping results confirmed the significance of the indirect effect, with a 95% CI [0.001, 0.066]. Therefore, Hypothesis 3a was also supported.

Regarding the path among perceived fake news seriousness, anger, and transnational fake news rebuttal, the results showed that perceived fake news seriousness had an indirect effect on transnational fake news rebuttal (B = 0.025). Bootstrapping results confirmed the significance of the indirect effect, with a 95% CI [0.002, 0.060]. Therefore, Hypothesis 3b was also supported.

### 4.3 Moderated mediation analysis

The results relating to Hypothesis 4a/b are reported in Table 3. The results demonstrated that the interaction between anxiety and information-seeking motivation negatively moderated transnational fake news rebuttal (B = −0.129, *p* < 0.05; see Table 3), which did not support Hypothesis 4a. Meanwhile, the results demonstrated that the interaction between anger and information-seeking motivation did not moderate transnational fake news rebuttal (B = 0.061, *p* > 0.05; see Table 3), which did not support Hypothesis 4b.

**Table 3:** Testing the moderated mediation effect of information-seeking motivation on transnational fake news rebuttal.

Predictors	Model 4 (anxiety)		Model 5 (anger)		Model 6 (rebuttal)	
	B (SE)	*t*	B(SE)	*t*	B (SE)	*t*
Age	0.067 (0.056)	1.186	0.050 (0.057)	0.874	0.124 (0.054)	2.295*
Gender	0.133 (0.112)	1.184	−0.109 (0.114)	−0.963	0.025 (0.107)	0.237
Education	−0.231 (0.090)	−2.564	−0.066 (0.091)	−0.724	0.054 (0.086)	0.624
Serious	0.188 (0.056)	3.354***	0.168 (0.057)	2.966**	0.122 (0.055)	2.231**
Anxiety	—	—	—	—	0.840 (0.319)	2.635**
Anger	—	—	—	—	−0.212 (0.298)	−0.712
information-seeking motivation	—	—	—	—	0.193 (0.052)	3.716***
Anxiety × ISM	—	—	—	—	−0.129 (0.056)	−2.325*
Anger × ISM	—	—	—	—	0.061 (0.053)	1.160
R2	0.057	—	0.036	—	0.172	—
F	4.626**	—	2.867*	—	6.917***	—

*N* = 309. Each column is a regression model that predicts the variable at the top of the column. ISM, information seeking motivation; *p < 0.05, **p < 0.01, ***p < 0.001.

We then plotted a slope to illustrate the relationship between anxiety and transnational fake news rebuttal, for low and high levels of information-seeking motivation, respectively. As Figure 2 shows, the slope of the relationship between anxiety and transnational fake news rebuttal with low information-seeking motivation was significant (B low information-seeking motivation = 0.253, *t* = 2.914, *p* < 0.05).

**Figure 2:**
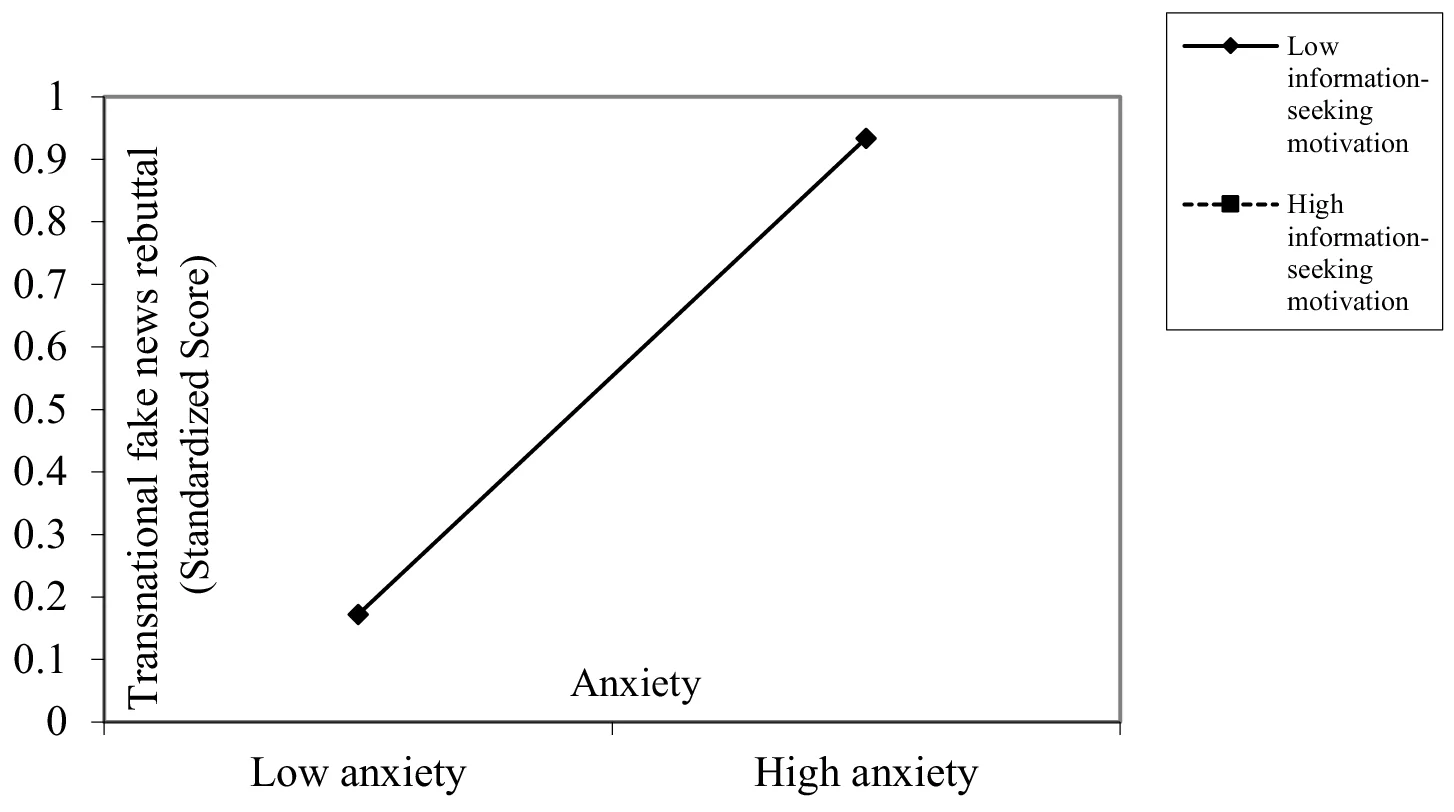
The interaction effect of anxiety and information-seeking motivation on transnational fake news rebuttal. High and low levels of anxiety and information-seeking motivation represent 1 standard deviation above and below the mean, respectively.

The results indicated that the index of moderated mediation on transnational fake news rebuttal was significant (B = −0.024, SE = 0.015, 95% CI = [−0.059, −0.001]). Specifically, the indirect effect of anxiety on rebuttal behavior through rebuttal intention was stronger among individuals with a low level of information-seeking motivation, and weaker or non-significant at higher levels. This finding suggests that, contrary to our hypothesis, information-seeking motivation negatively moderates the indirect effect of anxiety on rebuttal behavior. In other words, higher information-seeking motivation dampens the impact of anxiety on rebuttal responses. Thus, hypothesis 5 was not supported.

Summary results of moderation analysis are presented in Figure 3 below.

**Figure 3:**
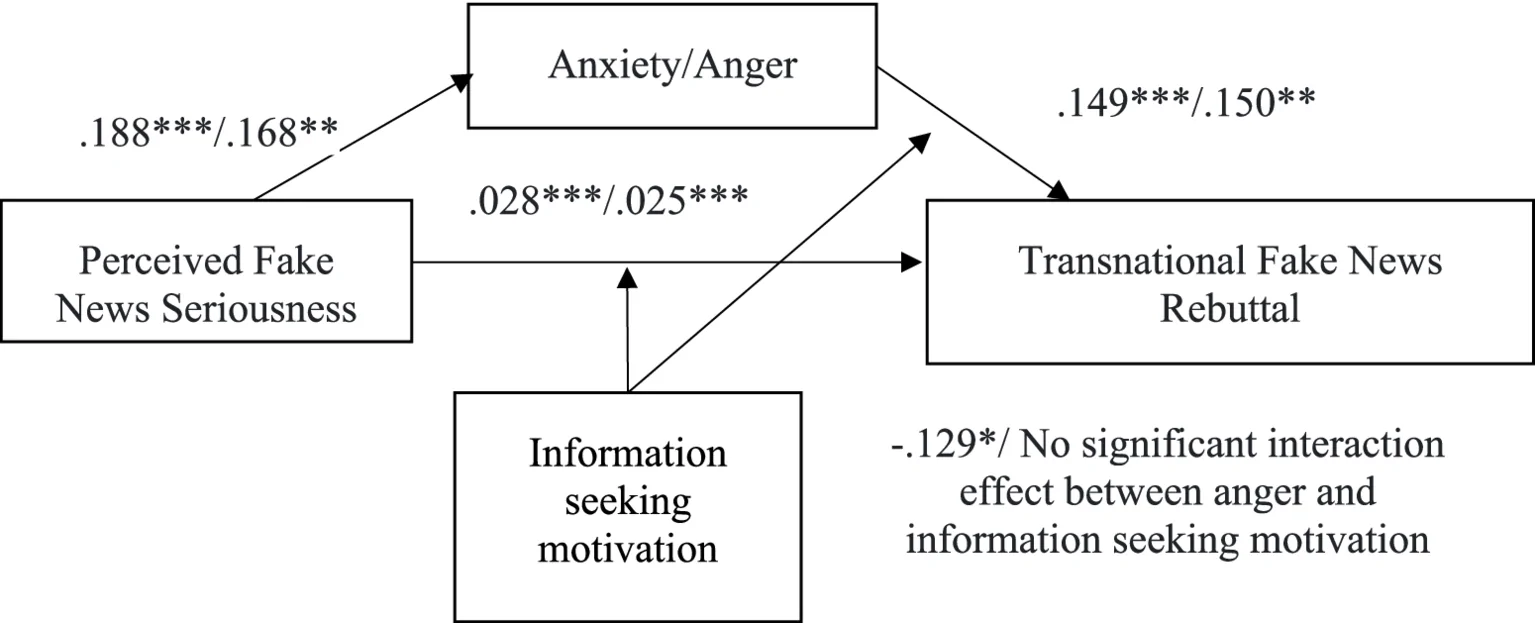
Summary results of the moderated mediation analysis.

## 5 Discussion

This study set out to investigate the psychological mechanisms underlying netizens’ rebuttal behaviors toward transnational fake news, with particular attention to the roles of perceived fake news seriousness, emotional responses, and -seeking motivation. Building on the third-person effects theory and emotion-centered frameworks, we proposed a moderated mediation model in which perceived issue seriousness for others triggers anxiety and anger, both of which promote rebuttal behaviors.

The foregoing results illustrate that both anxiety and anger mediate the relationship between perceived issue seriousness and rebuttal behavior. Among the two, anxiety emerged as a more consistent and robust mediator, indicating its stronger role in motivating users to engage in rebuttal. Contrary to expectations, information-seeking motivation was found to weaken, rather than enhance, the effect of anxiety on rebuttal, while its moderating effect on the association between anger and rebuttal behavior was not significant. These findings underscore the emotional and motivational dynamics driving user engagement with fake news, highlighting the importance of individual coping orientations in shaping disinformation responses.

### 5.1 Theoretical contributions

This study advances theoretical understanding of fake news rebuttal in several important ways.

First, this study extends third-person effect (TPE) research by showing how perceived influence on others can develop into a broader appraisal of fake news seriousness and subsequently motivate rebuttal behavior. Previous studies have established that individuals often perceive others as more vulnerable to fake news than themselves and that such third-person perceptions can shape support for information regulation, fact-checking, or corrective interventions (Jang and Kim, 2018; Corbu et al., 2020; Chung and Kim, 2021). However, less attention has been paid to the psychological mechanism through which perceived influence on others becomes translated into user-driven rebuttal behavior. By conceptualizing perceived fake news seriousness as a cognitive appraisal of informational harm, this study shows that third-person perceptions do not merely reflect a perceptual gap between the self and others. Rather, they may also activate users’ evaluations of the potential social, reputational, and communicative consequences of misinformation. This is particularly important in transnational contexts, where fake news may not only mislead individual audiences but also distort external publics’ interpretations of a country’s policies, actions, or social conditions. In this sense, the study enriches TPE research by connecting perceived influence on others with perceived seriousness, emotional arousal, and corrective communication.

Second, this study contributes to a more nuanced understanding of the affective mechanisms linking perceived fake news seriousness to rebuttal behavior. Instead of treating negative emotions as a general response to misinformation, this study distinguishes between anger and anxiety as discrete emotional pathways. Although both emotions were activated by perceived seriousness for others, anxiety proved to be a more robust and consistent predictor of rebuttal behavior than anger. This finding challenges assumptions in some prior studies that emphasize anger or outrage as the dominant mobilizing emotion in misinformation engagement and online sharing (Chuai and Zhao, 2022), and suggests that uncertainty-oriented emotional reactions deserve greater attention in research on corrective action in transnational digital contexts (Ghanem et al., 2020). One possible explanation is that although anger has mobilizing potential, it is often grounded in blame attribution and confrontational appraisal, which may lead to emotional expression, condemnation, or polarized responses rather than consistent rebuttal (Han et al., 2023). By contrast, under the TPE framework, anxiety is closely tied to the perceived vulnerability of external publics and uncertainty about misinformation consequences (Liu and Huang, 2020). Such anxiety may motivate users to verify information, clarify misleading claims, and reduce the perceived risk that others will be misled. In this sense, transnational fake news rebuttal is not driven only by outward hostility or retaliatory impulses, but may also arise from users’ concerns about uncertainty, reputational harm, and broader international misunderstanding.

Third, this study identifies information-seeking motivation as an important boundary condition in the emotion–behavior relationship. Based on the logic that TPE-related concerns about others may generate anxiety or anger, we initially expected that users with high information-seeking motivation would be more likely to transform such emotional arousal into purposeful corrective action. Contrary to this assumption, however, our findings reveal a significant negative moderation effect: the influence of anxiety on rebuttal behavior weakens as information-seeking motivation increases. This suggests that information-seeking motivation does not necessarily strengthen corrective action. Instead, it may redirect anxiety into more cautious or reflective coping strategies, such as verification, delay, further information search, or temporary disengagement (Sundar and Nass, 2000; Van Der Meer et al., 2023). In other words, rather than fueling action, high motivation may redirect anxiety into non-confrontational pathways.

Importantly, this moderation effect was found to be emotion-specific. While the anxiety–rebuttal pathway was weakened by higher information-seeking motivation, the anger–rebuttal pathway remained unaffected. This pattern suggests that anger, as a high-arousal and approach-oriented emotion, may activate a more direct mobilization pathway that is less contingent on users’ information-seeking orientation. By contrast, anxiety appears more sensitive to how individuals cope with uncertainty (Chaudhuri et al., 2025). This distinction is particularly salient in transnational fake news contexts, where misleading information is often embedded in ideological, nationalistic, or geopolitical frames and is therefore difficult for users to verify quickly. In such contexts, stronger information-seeking motivation may not immediately activate rebuttal behavior; instead, it may lead users toward verification, delayed response, or temporary disengagement.

Taken together, these findings suggest that emotional arousal does not translate into rebuttal behavior in a uniform way in transnational fake news contexts. Within the TPE framework, perceived seriousness for external publics may activate both anxiety and anger, but these emotions follow different behavioral routes. Anxiety-driven rebuttal is more contingent on how users cope with uncertainty, especially because transnational fake news often involves ambiguous sources, cross-cultural interpretations, and geopolitical frames that make rapid verification difficult. When information-seeking motivation redirects anxiety toward verification, delay, or temporary disengagement, its effect on rebuttal behavior may be weakened. Anger, by contrast, appears to motivate rebuttal through a more direct route that is less dependent on users’ information-seeking orientation. This emotion-specific pattern highlights the importance of examining not only whether users perceive transnational fake news as harmful to external publics but also how different emotions and motivations jointly shape their willingness to correct misleading narratives about their country.

### 5.2 Practical implications

The findings present several practical implications for platform governance, strategic communication, and media literacy initiatives in response to transnational fake news.

First, the study suggests that platform governance should move beyond content-centered correction and static fact-checking labels by incorporating users’ cognitive appraisals and emotional responses into debunking prompt design. Current systems often rely on behavioral history, such as prior clicks, topical interests, or engagement records, to recommend fact-checking content. However, our findings suggest that users who are not habitually engaged in news may still participate in rebuttal when emotionally activated. Therefore, content delivery and debunking systems should not only consider users’ behavioral profiles but also attend to signals of heightened emotional engagement and adopt more differentiated prompt strategies (Zhang et al., 2024). For users whose behavior suggests uncertainty-driven anxiety, such as repeated searching, delayed sharing, or engagement with verification-related content, platforms could prioritize structured, authoritative, and context-rich information. For users displaying signs of anger-driven rapid engagement, such as immediate liking, commenting, or sharing, prompts may first acknowledge users’ concerns before presenting corrective evidence. In this way, misinformation alerts would not simply flag inaccurate content but would also contextualize the perceived seriousness and emotional stakes of the issue, thereby encouraging constructive engagement rather than passive avoidance or hostile confrontation. At the same time, platforms should remain vigilant against the misuse of emotion-sensitive algorithms as tools of emotional manipulation, such as deliberately amplifying anger to increase engagement (McLoughlin and Brady, 2024; McLoughlin et al., 2024).

Second, the findings offer implications for the design of strategic communication to transnational fake news, which often carries symbolic, national, and geopolitical meanings. Unlike platform governance, which focuses on content delivery and prompt design, strategic communication concerns how communication actors explain contested issues, clarify misleading claims, and maintain credibility in cross-national information environments. Since public engagement with such misinformation is shaped not only by factual accuracy but also by users’ emotional states and motivational orientations (Martel et al., 2020), communication responses should avoid a purely corrective or one-size-fits-all approach. In addressing anger-driven responses, the goal should not be to amplify outrage, but to prevent emotionally charged rebuttal from escalating into polarization or hostile confrontation (Chuai and Zhao, 2022). Strategic communication efforts can help channel anger toward constructive correction by providing clear evidence, identifying the specific misleading claim, and maintaining a restrained and credible tone. For anxiety-driven responses, especially among users with stronger information-seeking motivation, repeatedly emphasizing the severity of misinformation may increase uncertainty and cognitive burden rather than encourage immediate rebuttal. In such cases, communication efforts should focus on reducing ambiguity by offering contextual explanations, transparent sources, and proportionate assessments of the issue. In this way, strategic communication can support public correction without encouraging excessive emotional mobilization or information overload.

Third, the findings also point to the cultural sensitivity of transnational fake news governance. Transnational fake news often involves culturally loaded symbols, geopolitical narratives, or cross-national comparisons. As a result, corrective communication should be carefully localized and contextualized rather than simply translated across languages (Folarin and Swiatek, 2025). The emotional resonance of a given message may vary depending on audiences’ prior attitudes, cultural backgrounds, and perceptions of intergroup or interstate relations. Therefore, communication strategies should consider not only linguistic translation, but also how different audience groups emotionally resonate with and cognitively process distinct cultural symbols.

Finally, the study also supports the necessity of incorporating emotion-oriented approaches into media literacy education. Traditional media literacy has focused on critical thinking, source evaluation, and fact-checking—skills rooted in cognitive processing. However, in today’s emotionally charged media environment, such cognitively oriented approaches may be insufficient. Users’ engagement with misinformation is frequently shaped by affective responses rather than analytical reasoning (Lühring et al., 2024). We suggest integrating emotional awareness and motivation-building into literacy programs, helping users recognize and manage emotions like anxiety and anger to better support intentional, adaptive rebuttal behavior.

## 6 Limitations and future research

There are several limitations to this study. First, the data consist solely of Chinese netizens, introducing a geographical limitation that potentially limits the generalizability of the findings. Information overload and fake news are global issues; users in different cultural contexts may adopt varying rebuttal mechanisms when countering transnational fake news (Pothitou et al., 2025). Therefore, future studies may consider broadening the data sources by incorporating netizens from diverse countries and regions, or by engaging in cross-cultural comparative analyses with existing research to assess the generalizability and cross-contextual validity of the findings. The second limitation concerns the fact that this study is cross-sectional in design, and causality is not confirmed. Thus, there is a need for future research to further investigate in a longitudinal method. Moreover, the current study focused on self-reported rebuttal intentions rather than actual behavior; further studies could employ an experimental design to evaluate users’ rebuttal behaviors (Ozturk et al., 2015).

## Data Availability

The raw data supporting the conclusions of this article will be made available by the authors, without undue reservation.

## References

[ref1] Bhagat S. Kim D. J. (2023). Examining users’ news sharing behaviour on social media: role of perception of online civic engagement and dual social influences. Behav. Inf. Technol. 42, 1194–1215. doi: 10.1080/0144929x.2022.2066019

[ref2] Brown S. P. Ganesan S. Challagalla G. (2001). Self-efficacy as a moderator of information-seeking effectiveness. J. Appl. Psychol. 86, 1043–1051. doi: 10.1037/0021-9010.86.5.1043, 11596798

[ref3] Carleton R. N. (2016). Into the unknown: a review and synthesis of contemporary models involving uncertainty. J. Anxiety Disord. 39, 30–43. doi: 10.1016/j.janxdis.2016.02.007, 26945765

[ref4] Chang C. (2021). Fake news: audience perceptions and concerted coping strategies. Digit. J. 9, 636–659. doi: 10.1080/21670811.2021.1923403

[ref5] Chaudhuri N. Gupta G. Popovic A. (2025). Do you believe it? Examining user engagement with fake news on social media platforms. Technol. Forecast. Soc. Change 212:123950. doi: 10.1016/j.techfore.2024.123950

[ref6] Chuai Y. Zhao J. (2022). Anger can make fake news viral online. Front. Phys. 10:970174. doi: 10.3389/fphy.2022.970174

[ref7] Chung M. Kim N. (2021). When I learn the news is false: how fact-checking information stems the spread of fake news via third-person perception. Hum. Commun. Res. 47, 1–24. doi: 10.1093/hcr/hqaa010

[ref8] CNNIC (2021). “The 47th Statistical Report on China’s Internet Development.(online) Available online at: https://www.cnnic.net.cn/NMediaFile/old_attach/P020210203334633480104.pdf (Accessed September 30, 2024)

[ref9] CNNIC (2026). “The 57th Statistical Report on China’s Internet Development (online) Available online at: https://www.cnnic.net.cn/NMediaFile/2026/0304/MAIN1772588317069TUXN3827X8.pdf (Accessed March 20, 2026).

[ref10] Coan T. G. Merolla J. L. Zechmeister E. J. Zizumbo-Colunga D. (2021). Emotional responses shape the substance of information seeking under conditions of threat. Polit. Res. Q. 74, 941–954. doi: 10.1177/1065912920949320

[ref11] Corbu N. Oprea D.-A. Negrea-Busuioc E. Radu L. (2020). They can’t fool me, but they can fool the others!‘third person effect and fake news detection. Eur. J. Commun. 35, 165–180. doi: 10.1177/0267323120903686

[ref12] Davison W. P. (1983). The third-person effect in communication. Public Opin. Q. 47, 1–15. doi: 10.1086/268763

[ref13] Deluggi N. Ashraf C. (2023). Liars, skeptics, cheerleaders: human rights implications of post-truth disinformation from state officials and politicians. Hum. Rights Rev. 24, 365–387. doi: 10.1007/s12142-023-00697-1

[ref14] Folarin M. Swiatek L. (2025). “Communicatively responding to global fake news crises: the importance of cultural sensitivity,” in Communicating in the Face of Global Crises, (Abingdon: Routledge), 184–200.

[ref15] Freiling I. Krause N. M. Scheufele D. A. Brossard D. (2023). Believing and sharing misinformation, fact-checks, and accurate information on social media: the role of anxiety during COVID-19. New Media Soc. 25, 141–162. doi: 10.1177/14614448211011451, 36620434 PMC9805917

[ref16] Ghanem B. Rosso P. Rangel F. (2020). An emotional analysis of false information in social media and news articles. ACM Trans. Internet Technol. 20, 1–18. doi: 10.1145/3381750

[ref17] Guess A. Nagler J. Tucker J. (2019). Less than you think: prevalence and predictors of fake news dissemination on Facebook. Sci. Adv. 5:eaau4586. doi: 10.1126/sciadv.aau4586, 30662946 PMC6326755

[ref18] Hameleers M. (2025). The visual nature of information warfare: the construction of partisan claims on truth and evidence in the context of wars in Ukraine and Israel/Palestine. J. Commun. 75, 90–100. doi: 10.1093/joc/jqae045

[ref19] Han J. Lee S. E. Cha M. (2023). The secret to successful evocative messages: anger takes the lead in information sharing over anxiety. Commun. Monogr. 90, 545–565. doi: 10.1080/03637751.2023.2236183

[ref20] Hayes A. F. (2012). PROCESS: A versatile Computational tool for Observed variable Mediation, Moderation, and Conditional process Modeling. Lawrence, KS: University of Kansas.

[ref21] Hayes A. F. Scharkow M. (2013). The relative trustworthiness of inferential tests of the indirect effect in statistical mediation analysis: does method really matter? Psychol. Sci. 24, 1918–1927. doi: 10.1177/0956797613480187 23955356

[ref22] Horberg E. J. Oveis C. Keltner D. (2011). Emotions as moral amplifiers: an appraisal tendency approach to the influences of distinct emotions upon moral judgment. Emotion Rev. 3, 237–244. doi: 10.1177/1754073911402384

[ref23] Horner C. G. Galletta D. Crawford J. Shirsat A. (2023). “Emotions: the unexplored fuel of fake news on social media,” in Fake news on the Internet, (Abingdon: Routledge), 147–174.

[ref24] Iqbal A. Shahzad K. Khan S. A. Chaudhry M. S. (2025). The relationship of artificial intelligence (AI) with fake news detection (FND): a systematic literature review. Glob. Knowl. Mem. Commun. 74, 1617–1637. doi: 10.1108/gkmc-07-2023-0264

[ref25] Jang S. M. Kim J. K. (2018). Third person effects of fake news: fake news regulation and media literacy interventions. Comput. Hum. Behav. 80, 295–302. doi: 10.1016/j.chb.2017.11.034

[ref9001] Johansson-Nogués E. Şimanschi E. (2023). Fabricating a war? Russian (dis)information on Ukraine. International Affairs 99, 2015–2036. doi: 10.1093/ia/iiad179

[ref26] Khan S. A. Shahzad K. Shabbir O. Iqbal A. (2022). Developing a framework for fake news diffusion control (FNDC) on digital media (DM): a systematic review 2010–2022. Sustainability 14:15287. doi: 10.3390/su142215287

[ref27] Knuutila A. Neudert L.-M. Howard P. N. (2022). Who is afraid of fake news?: modeling risk perceptions of misinformation in 142 countries. Harvard Kennedy School Misinformation Review 3:97. doi: 10.37016/mr-2020-97

[ref28] Lazarus R. S. (1991). Progress on a cognitive-motivational-relational theory of emotion. Am. Psychol. 46, 819–834. doi: 10.1037/0003-066x.46.8.819, 1928936

[ref29] Lee A. M. (2013). News audiences revisited: theorizing the link between audience motivations and news consumption. J. Broadcast. Electron. Media 57, 300–317. doi: 10.1080/08838151.2013.816712

[ref30] Lerner J. S. Keltner D. (2000). Beyond valence: toward a model of emotion-specific influences on judgement and choice. Cogn. Emot. 14, 473–493. doi: 10.1080/026999300402763

[ref31] Lerner J. S. Tiedens L. Z. (2006). Portrait of the angry decision maker: how appraisal tendencies shape anger's influence on cognition. J. Behav. Decis. Mak. 19, 115–137. doi: 10.1002/bdm.515

[ref32] Liao Q. V. Fu W.-T. (2013). Beyond the filter bubble: interactive effects of perceived threat and topic involvement on selective exposure to information, in: Proceedings of the SIGCHI conference on human factors in computing systems New York: Association for Computing Machinery. pp. 2359–2368

[ref33] Liu P. L. Huang L. V. (2020). Digital disinformation about COVID-19 and the third-person effect: examining the channel differences and negative emotional outcomes. Cyberpsychol. Behav. Soc. Netw. 23, 789–793. doi: 10.1089/cyber.2020.0363, 32757953

[ref34] Lo V.-H. Wei R. Su H. (2013). Self-efficacy, information-processing strategies, and acquisition of health knowledge. Asian J. Commun. 23, 54–67. doi: 10.1080/01292986.2012.725175

[ref35] Lühring J. Shetty A. Koschmieder C. Garcia D. Waldherr A. Metzler H. (2024). Emotions in misinformation studies: distinguishing affective state from emotional response and misinformation recognition from acceptance. Cogn. Res. Princ. Implic. 9:82. doi: 10.1186/s41235-024-00607-0, 39692779 PMC11656008

[ref36] Martel C. Pennycook G. Rand D. G. (2020). Reliance on emotion promotes belief in fake news. Cognitive Research: Principles and Implications, 5:47. doi: 10.1186/s41235-020-00252-3, 33026546 PMC7539247

[ref37] Mcloughlin K. L. Brady W. J. (2024). Human-algorithm interactions help explain the spread of misinformation. Curr. Opin. Psychol. 56:101770. doi: 10.1016/j.copsyc.2023.101770 38128169

[ref38] Mcloughlin K. L. Brady W. J. Goolsbee A. Kaiser B. Klonick K. Crockett M. (2024). Misinformation exploits outrage to spread online. Science 386, 991–996. doi: 10.1126/science.adl2829, 39607912

[ref39] Omar B. Al-Samarraie H. Alzahrani A. I. See Kee N. (2024). Unlocking young people’s engagement with online news: affective or cognitive? Online Inf. Rev. 48, 944–958. doi: 10.1108/oir-12-2022-0691

[ref40] Ozturk P. Li H. Sakamoto Y. (2015). Combating rumor spread on social media: the effectiveness of refutation and warning, in: 2015 48th Hawaii international conference on system sciences New York: IEEE. pp. 2406–2414.

[ref41] Panizza F. Ronzani P. Morisseau T. Mattavelli S. Martini C. (2023). How do online users respond to crowdsourced fact-checking? Hum. Soc. Sci. Commun. 10, 1–11. doi: 10.1057/s41599-023-02329-y

[ref42] Park H. S. Yun D. Choi H. J. Lee H. E. (2012). Perceived media bias and third person effect: comparisons of Americans, Korean Americans, and Koreans in the United States. Soc. Sci. J. 49, 139–149. doi: 10.1016/j.soscij.2011.08.009

[ref43] Paul B. Salwen M. B. Dupagne M. (2000). The third-person effect: a meta-analysis of the perceptual hypothesis. Mass Commun. Soc. 3, 57–85. doi: 10.1207/s15327825mcs0301_04

[ref44] Phiri C. (2025). Political Disinformation and the Law: Safeguarding Freedom of Expression in Europe. Abingdon: Routledge.

[ref45] Pothitou E. Perifanou M. Economides A. A. (2025). Cross-cultural perspectives on fake news: a comparative study of Instagram users in Greece and Portugal. Information 16:41. doi: 10.3390/info16010041

[ref46] Rampersad G. Althiyabi T. (2020). Fake news: acceptance by demographics and culture on social media. J. Inf. Technol. Polit. 17, 1–11. doi: 10.1080/19331681.2019.1686676

[ref47] Saeed M. Traub N. Nicolas M. Demartini G. Papotti P. (2022) Crowdsourced fact-checking at twitter: how does the crowd compare with experts? Proceedings of the 31st ACM international conference on information & knowledge management New York: Association for Computing Machinery. pp. 1736–1746

[ref48] Savolainen R. (2014). Emotions as motivators for information seeking: a conceptual analysis. Libr. Inf. Sci. Res. 36, 59–65. doi: 10.1016/j.lisr.2013.10.004

[ref49] Seib P. (2021). Information at war: Journalism, Disinformation, and modern Warfare. Hoboken: John Wiley & Sons.

[ref50] Shahzad K. Khan S. A. (2024). Relationship between new media literacy (NML) and web-based fake news epidemic control: a systematic literature review. Glob. Knowl. Mem. Commun. 73, 956–983. doi: 10.1108/gkmc-08-2022-0197

[ref51] Shahzad K. Khan S. A. Ahmad S. Iqbal A. (2022). A scoping review of the relationship of big data analytics with context-based fake news detection on digital media in data age. Sustainability 14:14365. doi: 10.3390/su142114365

[ref52] Shahzad K. Khan S. A. Iqbal A. Shabbir O. Latif M. (2023). Determinants of fake news diffusion on social media: a systematic literature review. Glob. Knowl. Mem. Commun. 74, 2069–2092. doi: 10.1108/gkmc-06-2023-0189

[ref53] Shen X. Helion C. Smith D. V. Murty V. P. (2024). Motivation as a lens for understanding information-seeking behaviors. J. Cogn. Neurosci. 36, 362–376. doi: 10.1162/jocn_a_02083, 37944120

[ref54] Steensen S. Ferrer-Conill R. Peters C. (2020). (against a) theory of audience engagement with news. Journalism Stud. 21, 1662–1680. doi: 10.1080/1461670x.2020.1788414

[ref55] Stenbjerre M. N. (1997). A Social Categorization Model of the Third Person Effect in Mass Communication. Ithaca, NY: Cornell University.

[ref58] Sun Y. Shen L. Pan Z. (2008). On the behavioral component of the third-person effect. Commun. Res. 35, 257–278. doi: 10.1177/0093650207313167

[ref59] Sun Y. Xie J. (2024). Who shares misinformation on social media? A meta-analysis of individual traits related to misinformation sharing. Comput. Hum. Behav. 158:108271. doi: 10.1016/j.chb.2024.108271

[ref60] Sundar S. S. Nass C. (2000). Source orientation in human-computer interaction: programmer, networker, or independent social actor. Commun. Res. 27, 683–703. doi: 10.1177/009365000027006001

[ref61] Tandoc E. C. Lim Z. W. Ling R. (2018). Defining “fake news” a typology of scholarly definitions. Digit. J. 6, 137–153. doi: 10.1080/21670811.2017.1360143

[ref9002] Van Der Meer T. G. Hameleers M. Ohme J. (2023). Can fighting misinformation have a negative spillover effect? How warnings for the threat of misinformation can decrease general news credibility. Journalism Studies 24, 803–823. doi: 10.1080/1461670X.2023.2187652, 29590045

[ref63] Vasu N. Ang B. Teo T.-A. Jayakumar S. Raizal M. Ahuja J. (2018). Fake News: National Security in the Post-Truth Era. Singapore: S. Rajaratnam School of International Studies.

[ref64] Vosoughi S. Roy D. Aral S. (2018). The spread of true and false news online. Science 359, 1146–1151. doi: 10.1126/science.aap9559, 29590045

[ref65] Waisbord S. (2018). Truth is what happens to news: on journalism, fake news, and post-truth. J. Stud. 19, 1866–1878. doi: 10.1080/1461670X.2018.1492881

[ref66] Wardle C. Derakhshan H. (2017). Information Disorder: Toward an Interdisciplinary Framework for Research and Policymaking. Strasbourg: Council of Europe.

[ref67] Watson D. Clark L. A. Tellegen A. (1988). Development and validation of brief measures of positive and negative affect: the PANAS scales. J. Pers. Soc. Psychol. 54, 1063–1070. doi: 10.1037/0022-3514.54.6.1063, 3397865

[ref9006] White A. H. (1997). Considering Interacting Factors in the Third-Person Effect: Argument Strength and Social Distance. Journalism & Mass Communication Quarterly 74, 557–564. doi: 10.1177/107769909707400309

[ref68] Xiao X. (2024). Let’s verify and rectify! Examining the nuanced influence of risk appraisal and norms in combatting misinformation. New Media Soc. 26, 3786–3809. doi: 10.1177/14614448221104948

[ref70] Zhang S. Zhang Y. Li J. Ni Z. Liu Z. (2024). Heart or mind? The impact of congruence on the persuasiveness of cognitive versus affective appeals in debunking messages on social media during public health crises. Comput. Hum. Behav. 154:108136. doi: 10.1016/j.chb.2024.108136

[ref71] Zhou Y. Shen L. (2024). Processing of misinformation as motivational and cognitive biases. Front. Psychol. 15:1430953. doi: 10.3389/fpsyg.2024.1430953, 39282675 PMC11393549

